# P-514. Emerging Dominant Variants of Human Metapneumovirus in the United States

**DOI:** 10.1093/ofid/ofaf695.729

**Published:** 2026-01-11

**Authors:** Lora Pless, Lambodhar Damodaran, Raymond Pomponio, Rose Patrick, Marrisa P Griffifth, Sara Walters, Kady D Waggle, Atalia Pleskovitch, Vatsala Srinivasa, Cole Varela, Leah Goldstein, Heidi L Moline, Lee Harrison, John Barton, Louise Moncla, Marian G Michaels, John Williams, Anna Wang-Erickson

**Affiliations:** University of Pittsburgh, Pittsburgh, Pennsylvania; University of Pennsylvania, Philadelphia, Pennsylvania; University of Pittsburgh, Pittsburgh, Pennsylvania; University of Pittsburgh, Pittsburgh, Pennsylvania; University of Pittsburgh, Pittsburgh, Pennsylvania; University of Pittsburgh, Pittsburgh, Pennsylvania; University of Pittsburgh, Pittsburgh, Pennsylvania; University of Pittsburgh, Pittsburgh, Pennsylvania; University of Pittsburgh, Pittsburgh, Pennsylvania; University of Pittsburgh, Pittsburgh, Pennsylvania; Centers for Disease Control and Prevention, Atlanta, Georgia; US-CDC, Atlanta, Georgia; University of Pittsburgh, Pittsburgh, Pennsylvania; University of Pittsburgh, Pittsburgh, Pennsylvania; University of Pennsylvania, Philadelphia, Pennsylvania; University of Pittsburgh/ CHP, Pittsburgh, Pennsylvania; University of Wisconsin, Madison, Wisconsin; University of Pittsburgh, Pittsburgh, Pennsylvania

## Abstract

**Background:**

Human metapneumovirus (HMPV) causes acute respiratory disease worldwide and is the second leading cause of lower respiratory infection and hospitalization in young children in the US. There is no licensed vaccine or therapeutic. HMPV mutates rapidly; however, the specific genomic elements that explain strain dominance remain undefined because there is limited routine genomic surveillance of HMPV.

**Methods:**

Using HMPV-positive nasal swab specimens prospectively collected from children aged < 18 years seeking medical care for acute respiratory illness in Pittsburgh, PA from 2017-2020, we performed whole genome sequencing (Illumina) on 219 specimens, followed by *de novo* genome assembly (SPAdes) and quality control screening (≥ 95% genome coverage at ≥10× depth).

**Results:**

Only A2, B1, and B2 subgroups were detected; the dominant subgroup varied between seasons. The A1 subgroup was not detected, consistent with previous observations that it may be extinct. In A2 viruses, an in-frame 111- or 180-nucleotide (nt) insertion that nearly duplicates the preceding flanking region in the 660-nt G gene (encodes the attachment protein) was commonly detected. In B2 viruses, smaller in-frame insertions were also frequently detected in the same location of the G gene. Each insertion length formed a distinct phylogenetic clade. The insertions are in the C-terminal region of the ectodomain and contain positively-charged residues or predicted O-glycosylation sites. Sanger sequencing confirmed our genome assembly methods could accurately detect the insertions.

**Conclusion:**

To our knowledge, this is the first detection of such insertion variants in the US. Though the B2 insertion variants have not been reported elsewhere, the A2 180-nt variant was first reported in Japan and Spain in 2017, and the 111-nt variant was reported to be dominant in Yokohama, Japan by 2018. Future work will determine whether these insertions are common in other US cities and investigate any transmission advantage of these and other mutations in the genome.

**Disclosures:**

Lora Pless, PhD, Astra Zeneca - Icosavax: Grant/Research Support Raymond Pomponio, MS, Aidance Scientific: Advisor/Consultant|UE Life Sciences: Advisor/Consultant Lee Harrison, MD, GSK: Advisor/Consultant|Merck: Board Member|Pfizer: Advisor/Consultant|Sanofi: Advisor/Consultant John Barton, PhD, Moderna: Stocks/Bonds (Public Company) Marian G. Michaels, MD, MPH, Merck: Grant/Research Support

